# In this month’s Bulletin 

**DOI:** 10.2471/BLT.26.000326

**Published:** 2026-03-01

In the editorial section, Katharine Wright and Katherine Littler (138) introduce this month’s special issue on the ethics of climate-related health research. Gabrielle Samuel (139) discusses policy options on a sustainable path for clinical trials and Jerome Amir Singh (140) examines values and trade-offs in this field.

Alejandro Daly talks to Gary Humphreys (143–144) about his work in the global movement for clean air and climate justice. 

## Brazil

### Tuberculosis in children and adolescents

Antônio Candido Paiva Figueiredo dos Santos et al. (145–154) compare notifications before and after the COVID-19 pandemic.

## Colombia, France, India, United States of America

### Life in hot cities

Alaha Nasari and Sammer Marzouk (163–167) describe extreme heat adaptation measures.

## Greece

### How to prepare for the coming flood

Varvara A Mouchtouri et al. (175–183) analyse response measures.

## Uganda, Zambia

### Measuring the health sector’s environmental impact

Rachel Lisa King et al. (203–205) propose ways to quantify and reduce emissions.

## Viet Nam, Zimbabwe

### Community engagement in climate and health research

Yasna Palmeiro-Silva et al. (206–208) advocate for wider participation.

## WHO African Region

### Babies’ access to human milk

Emily M Njuguna et al. (194–196) point to improved neonatal outcomes.

## Global

### Setting ethical research priorities

Bridget Pratt et al. (155–162) present WHO’s criteria.

Neil Singh Bedi et al. (197–199) list contributions made by decision science.

### Health justice and climate change

Kyle Ferguson et al. (184–193) assign responsibilities in adaptation research.

Hiroaki Matsuura and Takefumi Ueno (200–202) explore insurance company mandates.

### Regulation of climate change and health research

Godwin Pancras et al. (168–174) detail roles of ethics committees.

